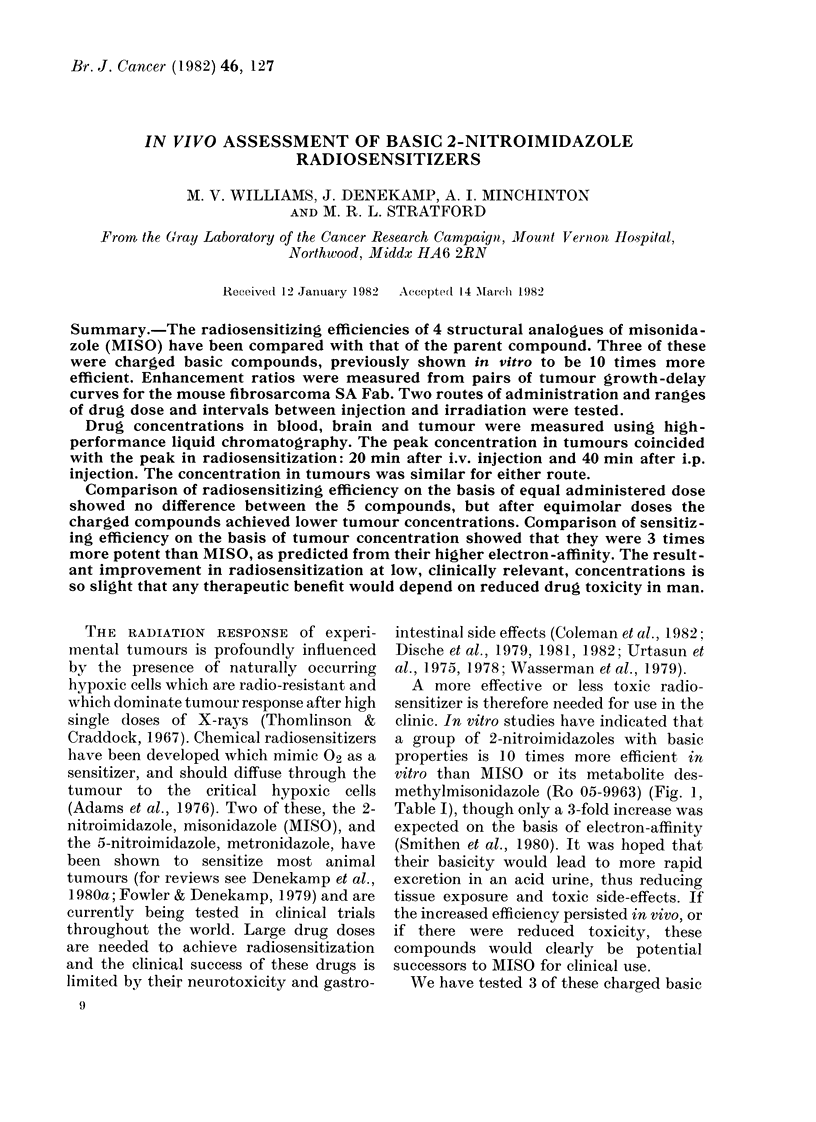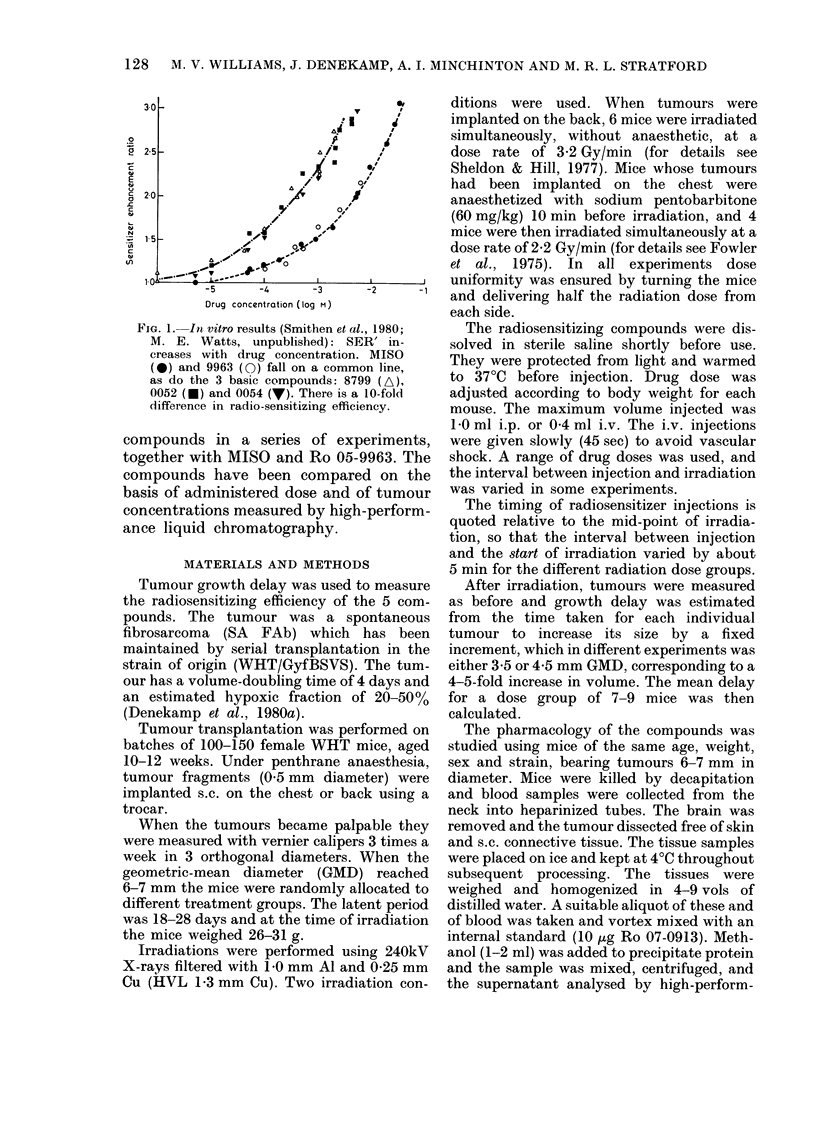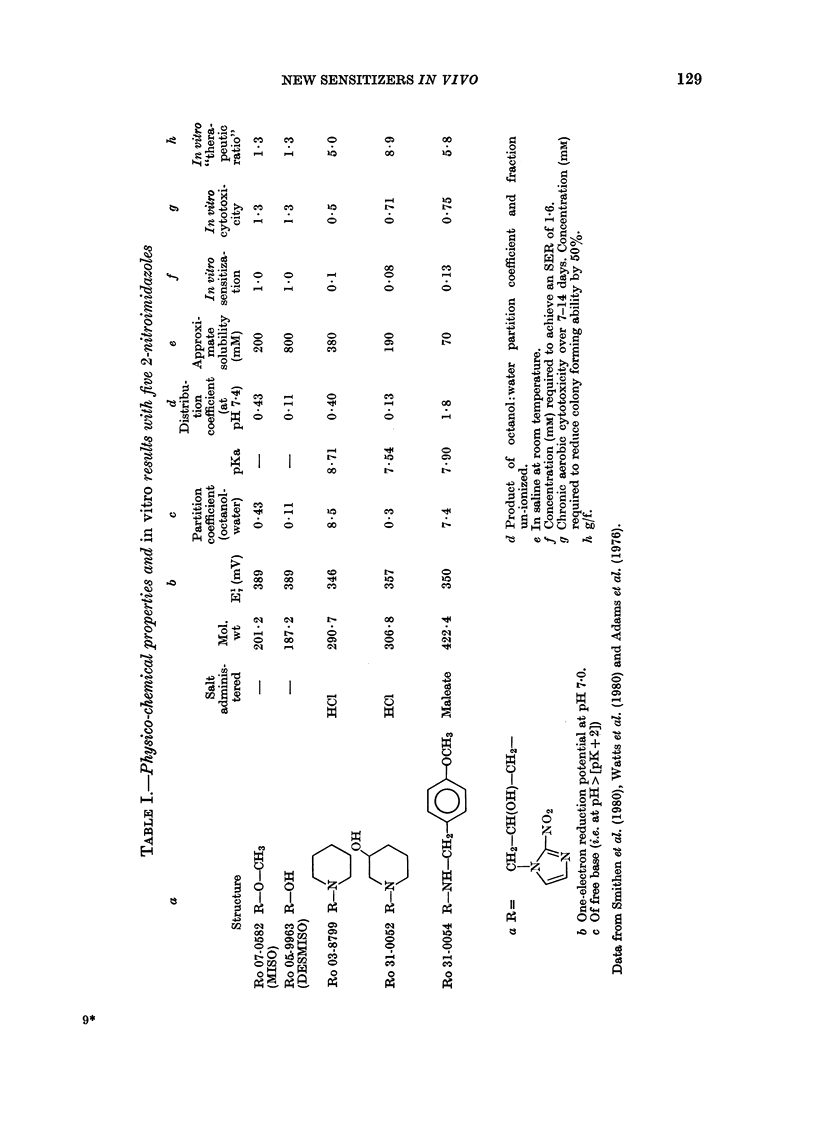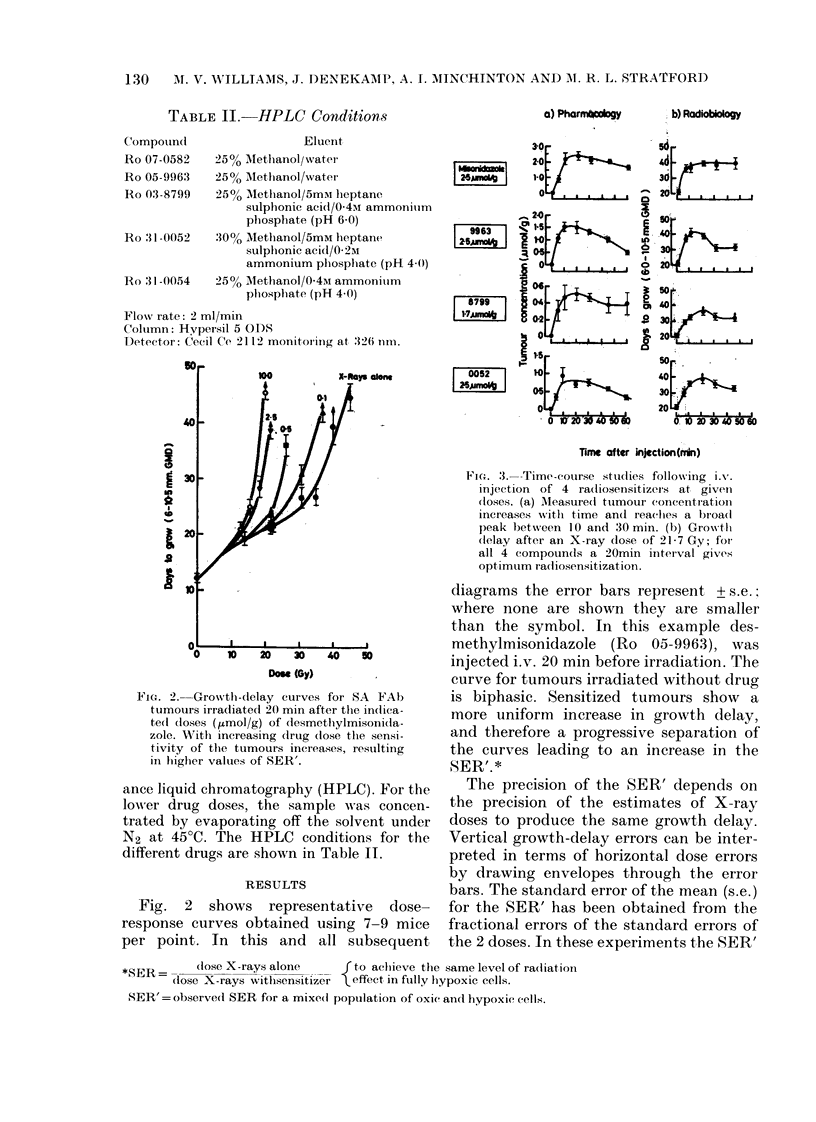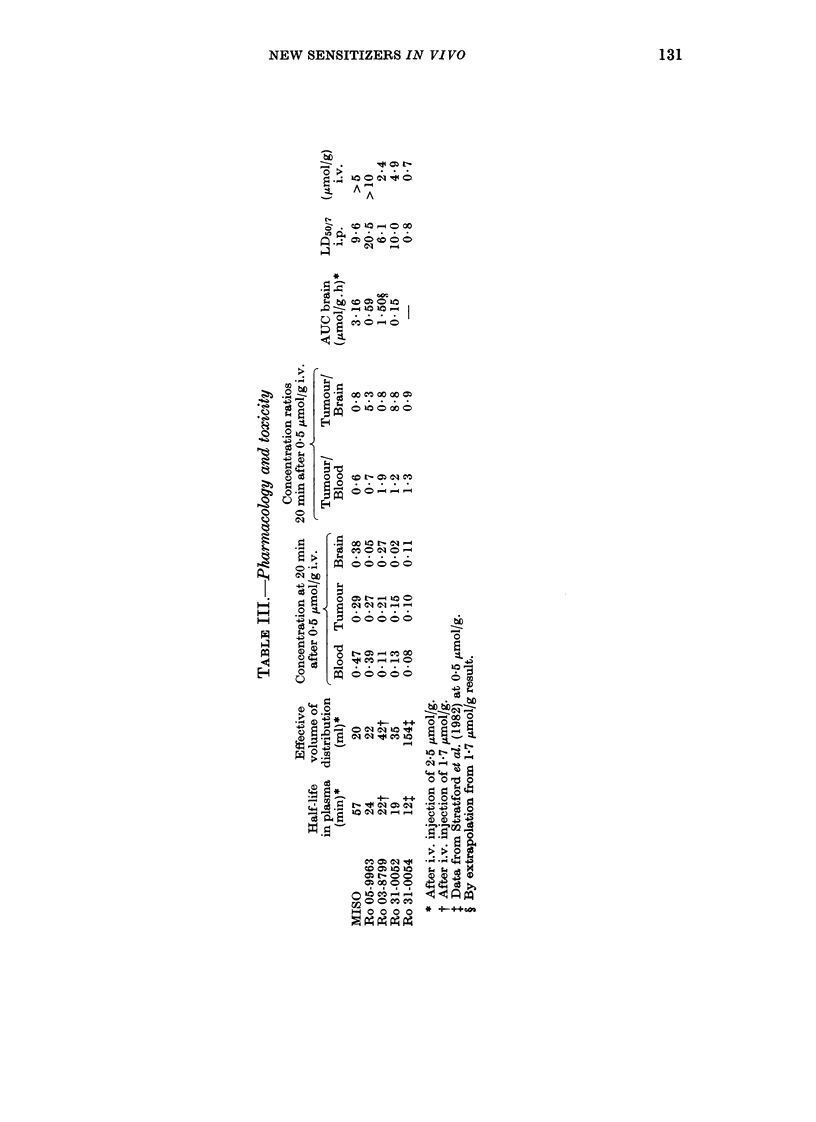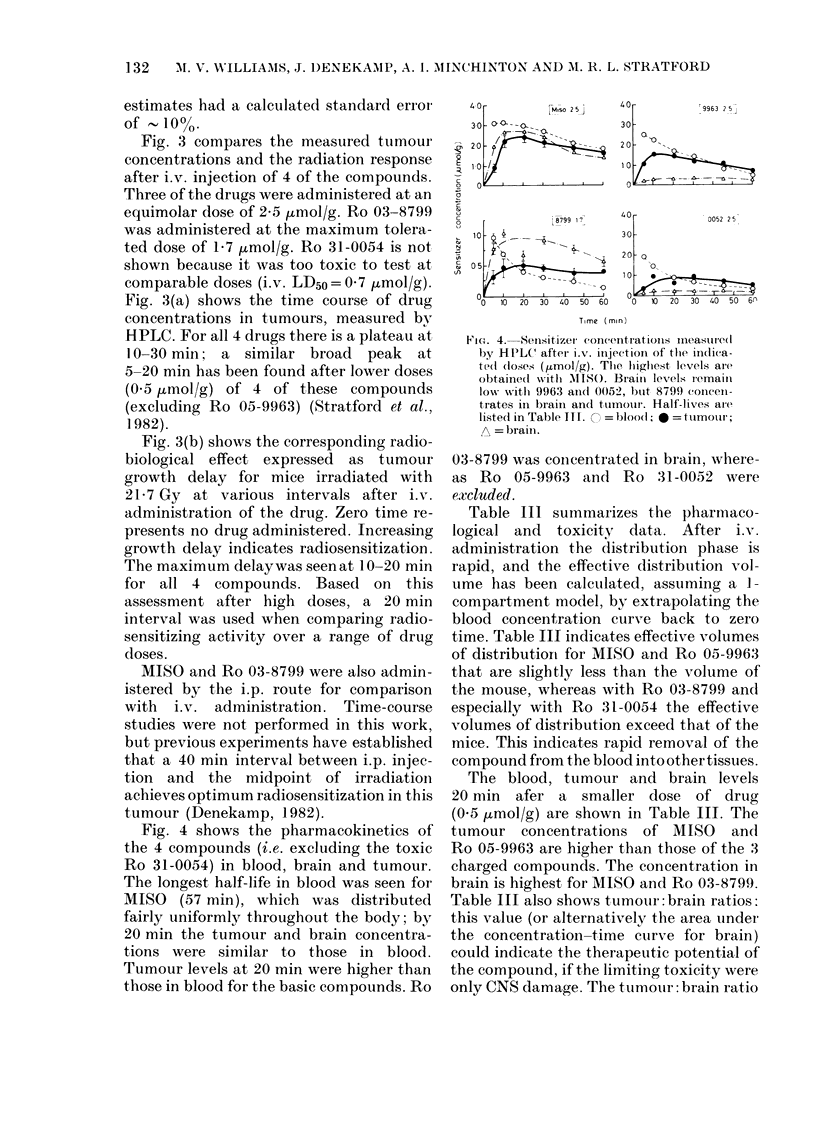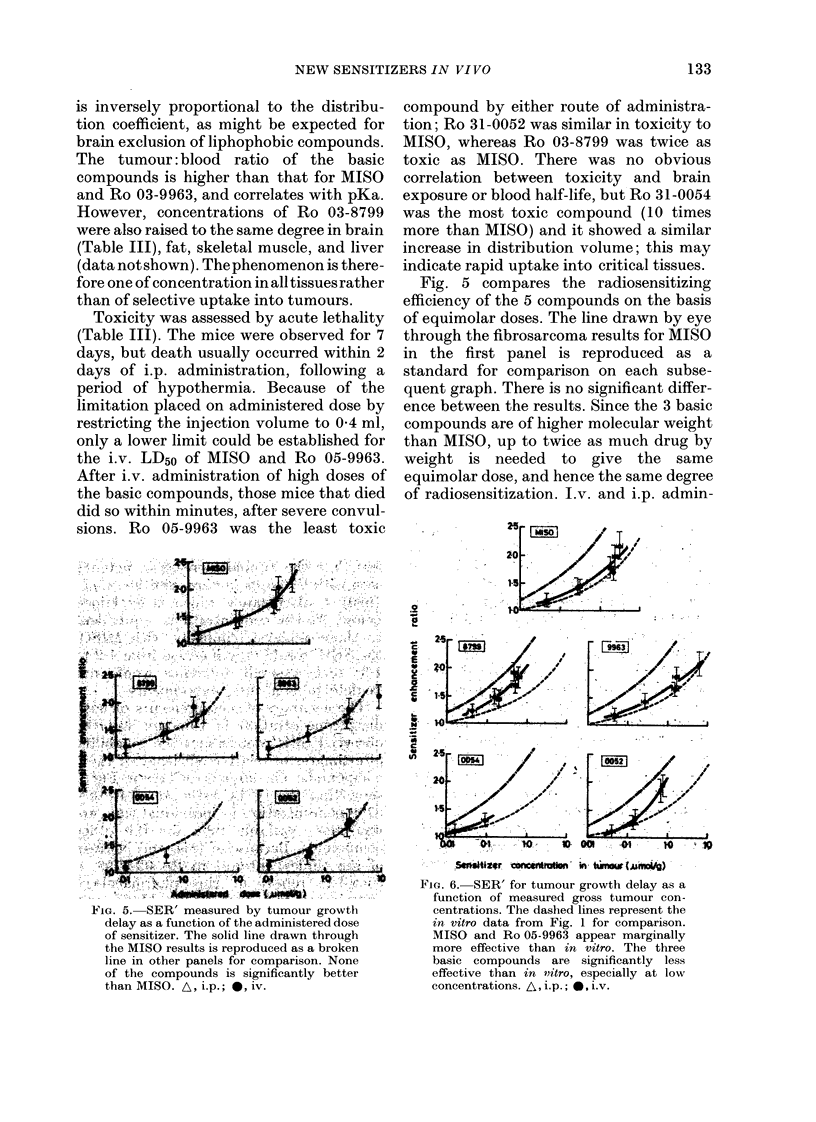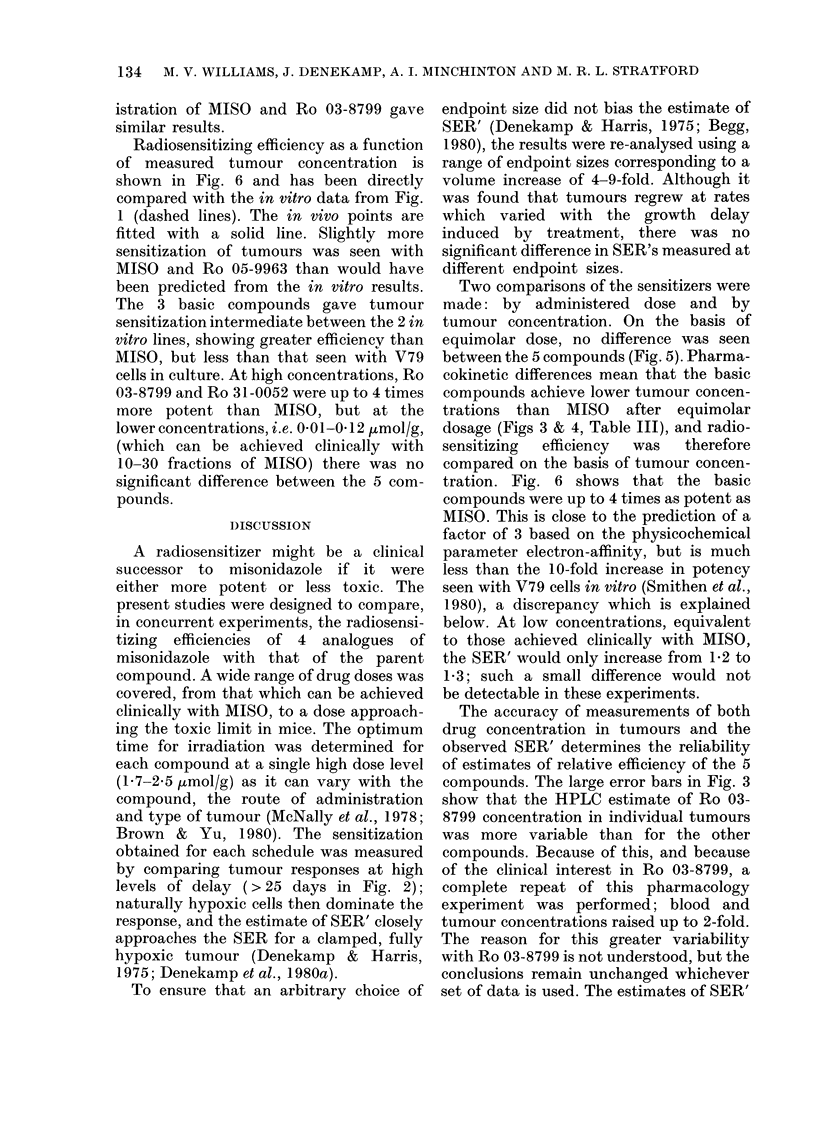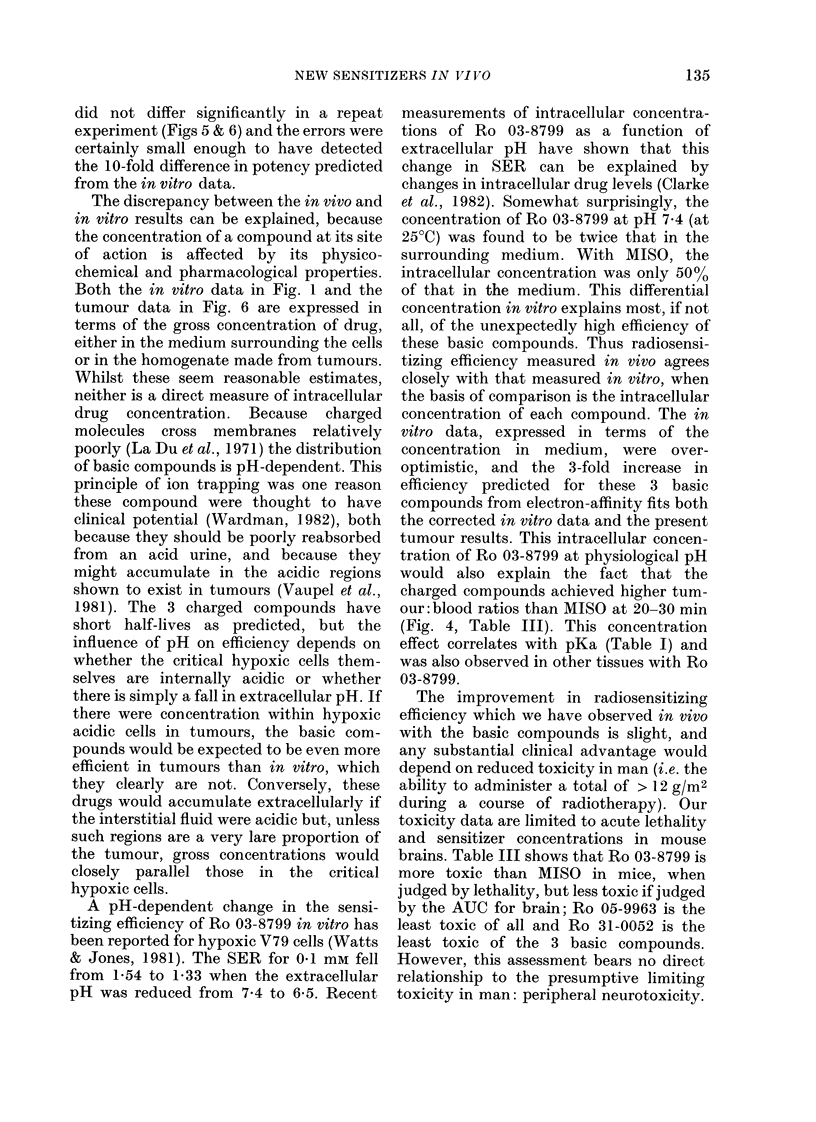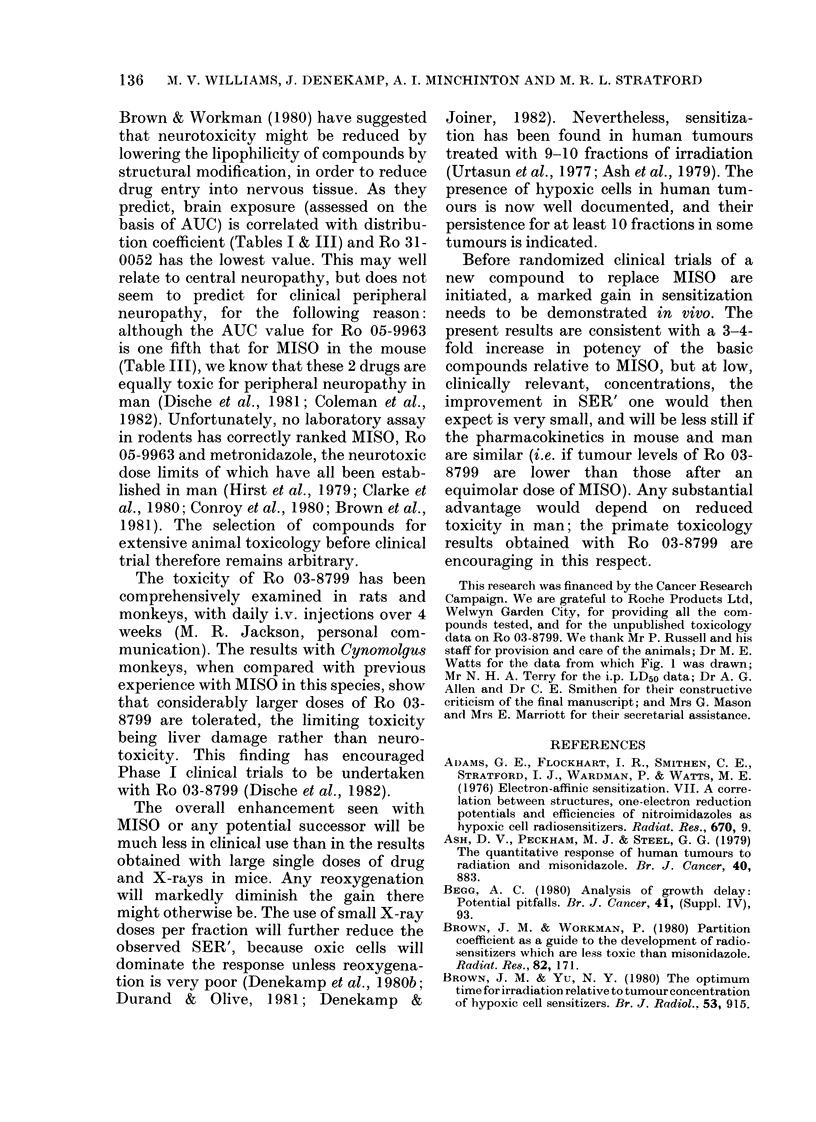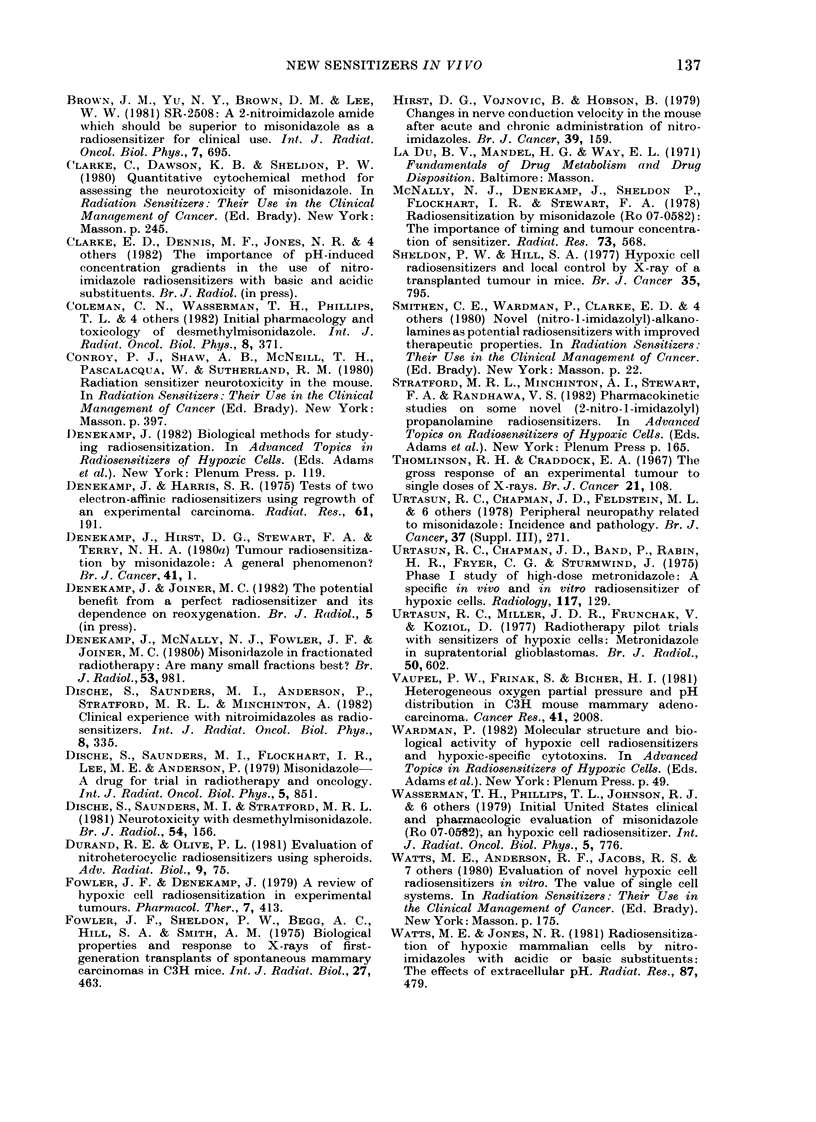# In vivo assessment of basic 2-nitroimidazole radiosensitizers.

**DOI:** 10.1038/bjc.1982.174

**Published:** 1982-07

**Authors:** M. V. Williams, J. Denekamp, A. I. Minchinton, M. R. Stratford

## Abstract

The radiosensitizing efficiencies of 4 structural analogues of misonidazole (MISO) have been compared with that of the parent compound. Three of these were charged basic compounds, previously shown in vitro to be 10 times more efficient. Enhancement ratios were measured from pairs of tumour growth-delay curves for the mouse fibrosarcoma SA Fab. Two routes of administration and ranges of drug dose and intervals between injection and irradiation were tested. Drug concentrations in blood, brain and tumor were measured using high-performance liquid chromatography. The peak concentration in tumours coincided with the peak in radiosensitization: 20 min after i.v. injection and 40 min after i.p. injection. The concentration in tumours was similar for either route. Comparison of radiosensitizing efficiency on the basic of equal administered dose showed no difference between the 5 compounds, but after equimolar doses the charged compounds achieved lower tumour concentrations. Comparison of sensitizing efficiency on the basis of tumour concentration showed that they were 3 times more potent than MISO, as predicted from their higher electron-affinity. The resultant improvement in radiosensitization at low, clinically relevant, concentrations is so slight that any therapeutic benefit would depend on reduced drug toxicity in man.


*Br. J. Cancer* (1982) **46**, 127

### *IN VIVO* ASSESSMENT OF BASIC 2-NITROIMIDAZOLE RADIOSENSITIZERS

M. V. WILLIAMS, J. DENEKAMP, A. I. MINCHINTON AND M. R. L. STRATFORD

*From the Gray Laboratory of the Cancer Research Campaign, Mount Vernon Hospital, Northwood, Middx HA6 2RN*

Received 12 January 1982 Accepted 14 March 1982

**Summary.—The radiosensitizing efficiencies of 4 structural analogues of misonidazole (MISO) have been compared with that of the parent compound. Three of these were charged basic compounds, previously shown *in vitro* to be 10 times more efficient. Enhancement ratios were measured from pairs of tumour growth-delay curves for the mouse fibrosarcoma SA Fab. Two routes of administration and ranges of drug dose and intervals between injection and irradiation were tested.**

**Drug concentrations in blood, brain and tumour were measured using high-performance liquid chromatography. The peak concentration in tumours coincided with the peak in radiosensitization: 20 min after i.v. injection and 40 min after i.p. injection. The concentration in tumours was similar for either route.**

**Comparison of radiosensitizing efficiency on the basis of equal administered dose showed no difference between the 5 compounds, but after equimolar doses the charged compounds achieved lower tumour concentrations. Comparison of sensitizing efficiency on the basis of tumour concentration showed that they were 3 times more potent than MISO, as predicted from their higher electron-affinity. The resultant improvement in radiosensitization at low, clinically relevant, concentrations is so slight that any therapeutic benefit would depend on reduced drug toxicity in man.**

THE RADIATION RESPONSE of experimental tumours is profoundly influenced by the presence of naturally occurring hypoxic cells which are radio-resistant and which dominate tumour response after high single doses of X-rays (Thomlinson & Craddock, 1967). Chemical radiosensitizers have been developed which mimic $O_2$ as a sensitizer, and should diffuse through the tumour to the critical hypoxic cells (Adams *et al.*, 1976). Two of these, the 2-nitroimidazole, misonidazole (MISO), and the 5-nitroimidazole, metronidazole, have been shown to sensitize most animal tumours (for reviews see Denekamp *et al.*, 1980*a*; Fowler & Denekamp, 1979) and are currently being tested in clinical trials throughout the world. Large drug doses are needed to achieve radiosensitization and the clinical success of these drugs is limited by their neurotoxicity and gastro-intestinal side effects (Coleman *et al.*, 1982; Dische *et al.*, 1979, 1981, 1982; Urtasun *et al.*, 1975, 1978; Wasserman *et al.*, 1979).

A more effective or less toxic radiosensitizer is therefore needed for use in the clinic. *In vitro* studies have indicated that a group of 2-nitroimidazoles with basic properties is 10 times more efficient *in vitro* than MISO or its metabolite desmethylmisonidazole (Ro 05-9963) (Fig. 1, Table I), though only a 3-fold increase was expected on the basis of electron-affinity (Smithen *et al.*, 1980). It was hoped that their basicity would lead to more rapid excretion in an acid urine, thus reducing tissue exposure and toxic side-effects. If the increased efficiency persisted *in vivo*, or if there were reduced toxicity, these compounds would clearly be potential successors to MISO for clinical use.

We have tested 3 of these charged basic

FIG. 1.—*In vitro* results (Smithen *et al.*, 1980; M. E. Watts, unpublished): SER′ increases with drug concentration. MISO (●) and 9963 (○) fall on a common line, as do the 3 basic compounds: 8799 (△), 0052 (■) and 0054 (▼). There is a 10-fold difference in radio-sensitizing efficiency.

compounds in a series of experiments, together with MISO and Ro 05-9963. The compounds have been compared on the basis of administered dose and of tumour concentrations measured by high-performance liquid chromatography.

MATERIALS AND METHODS

Tumour growth delay was used to measure the radiosensitizing efficiency of the 5 compounds. The tumour was a spontaneous fibrosarcoma (SA FAb) which has been maintained by serial transplantation in the strain of origin (WHT/GyfBSVS). The tumour has a volume-doubling time of 4 days and an estimated hypoxic fraction of 20–50% (Denekamp *et al.*, 1980*a*).

Tumour transplantation was performed on batches of 100–150 female WHT mice, aged 10–12 weeks. Under penthrane anaesthesia, tumour fragments (0·5 mm diameter) were implanted s.c. on the chest or back using a trocar.

When the tumours became palpable they were measured with vernier calipers 3 times a week in 3 orthogonal diameters. When the geometric-mean diameter (GMD) reached 6–7 mm the mice were randomly allocated to different treatment groups. The latent period was 18–28 days and at the time of irradiation the mice weighed 26–31 g.

Irradiations were performed using 240kV X-rays filtered with 1·0 mm Al and 0·25 mm Cu (HVL 1·3 mm Cu). Two irradiation conditions were used. When tumours were implanted on the back, 6 mice were irradiated simultaneously, without anaesthetic, at a dose rate of 3·2 Gy/min (for details see Sheldon & Hill, 1977). Mice whose tumours had been implanted on the chest were anaesthetized with sodium pentobarbitone (60 mg/kg) 10 min before irradiation, and 4 mice were then irradiated simultaneously at a dose rate of 2·2 Gy/min (for details see Fowler *et al.*, 1975). In all experiments dose uniformity was ensured by turning the mice and delivering half the radiation dose from each side.

The radiosensitizing compounds were dissolved in sterile saline shortly before use. They were protected from light and warmed to 37°C before injection. Drug dose was adjusted according to body weight for each mouse. The maximum volume injected was 1·0 ml i.p. or 0·4 ml i.v. The i.v. injections were given slowly (45 sec) to avoid vascular shock. A range of drug doses was used, and the interval between injection and irradiation was varied in some experiments.

The timing of radiosensitizer injections is quoted relative to the mid-point of irradiation, so that the interval between injection and the *start* of irradiation varied by about 5 min for the different radiation dose groups.

After irradiation, tumours were measured as before and growth delay was estimated from the time taken for each individual tumour to increase its size by a fixed increment, which in different experiments was either 3·5 or 4·5 mm GMD, corresponding to a 4–5-fold increase in volume. The mean delay for a dose group of 7–9 mice was then calculated.

The pharmacology of the compounds was studied using mice of the same age, weight, sex and strain, bearing tumours 6–7 mm in diameter. Mice were killed by decapitation and blood samples were collected from the neck into heparinized tubes. The brain was removed and the tumour dissected free of skin and s.c. connective tissue. The tissue samples were placed on ice and kept at 4°C throughout subsequent processing. The tissues were weighed and homogenized in 4–9 vols of distilled water. A suitable aliquot of these and of blood was taken and vortex mixed with an internal standard (10 μg Ro 07-0913). Methanol (1–2 ml) was added to precipitate protein and the sample was mixed, centrifuged, and the supernatant analysed by high-perform-

TABLE I.—*Physico-chemical properties and* in vitro *results with five 2-nitroimidazoles*

| *a* Structure | | Salt administered | Mol. wt | *b* $E^1_7$ (mV) | *c* Partition coefficient (octanol-water) | pKa | *d* Distribution coefficient (at pH 7·4) | *e* Approximate solubility (mM) | *f* *In vitro* sensitization | *g* *In vitro* cytotoxicity | *h* *In vitro* "therapeutic ratio" |
|---|---|---|---|---|---|---|---|---|---|---|---|
| Ro 07-0582 (MISO) | R—O—CH3 | — | 201·2 | 389 | 0·43 | — | 0·43 | 200 | 1·0 | 1·3 | 1·3 |
| Ro 05-9963 (DESMISO) | R—OH | — | 187·2 | 389 | 0·11 | — | 0·11 | 800 | 1·0 | 1·3 | 1·3 |
| Ro 03-8799 | R—N  | HCl | 290·7 | 346 | 8·5 | 8·71 | 0·40 | 380 | 0·1 | 0·5 | 5·0 |
| Ro 31-0052 | R—N  | HCl | 306·8 | 357 | 0·3 | 7·54 | 0·13 | 190 | 0·08 | 0·71 | 8·9 |
| Ro 31-0054 | R—NH—CH2—  —OCH3 | Maleate | 422·4 | 350 | 7·4 | 7·90 | 1·8 | 70 | 0·13 | 0·75 | 5·8 |

*a* R=

*b* One-electron reduction potential at pH 7·0.

*c* Of free base (*i.e.* at pH > [pK + 2])

*d* Product of octanol:water partition coefficient and fraction un-ionized.

*e* In saline at room temperature.

*f* Concentration (mM) required to achieve an SER of 1·6.

*g* Chronic aerobic cytotoxicity over 7–14 days. Concentration (mM) required to reduce colony forming ability by 50%.

*h* g/f.

Data from Smithen *et al.* (1980), Watts *et al.* (1980) and Adams *et al.* (1976).

TABLE II.—*HPLC Conditions*

| Compound | Eluent |
|---|---|
| Ro 07-0582 | 25% Methanol/water |
| Ro 05-9963 | 25% Methanol/water |
| Ro 03-8799 | 25% Methanol/5mM heptane sulphonic acid/0·4M ammonium phosphate (pH 6·0) |
| Ro 31-0052 | 30% Methanol/5mM heptane sulphonic acid/0·2M ammonium phosphate (pH 4·0) |
| Ro 31-0054 | 25% Methanol/0·4M ammonium phosphate (pH 4·0) |

Flow rate: 2 ml/min

Column: Hypersil 5 ODS

Detector: Cecil Ce 2112 monitoring at 326 nm.

FIG. 2.—Growth-delay curves for SA FAb tumours irradiated 20 min after the indicated doses (μmol/g) of desmethylmisonidazole. With increasing drug dose the sensitivity of the tumours increases, resulting in higher values of SER′.

ance liquid chromatography (HPLC). For the lower drug doses, the sample was concentrated by evaporating off the solvent under $N_2$ at 45°C. The HPLC conditions for the different drugs are shown in Table II.

RESULTS

Fig. 2 shows representative dose–response curves obtained using 7–9 mice per point. In this and all subsequent

FIG. 3.—Time-course studies following i.v. injection of 4 radiosensitizers at given doses. (a) Measured tumour concentration increases with time and reaches a broad peak between 10 and 30 min. (b) Growth delay after an X-ray dose of 21·7 Gy; for all 4 compounds a 20min interval gives optimum radiosensitization.

diagrams the error bars represent ± s.e.; where none are shown they are smaller than the symbol. In this example desmethylmisonidazole (Ro 05-9963), was injected i.v. 20 min before irradiation. The curve for tumours irradiated without drug is biphasic. Sensitized tumours show a more uniform increase in growth delay, and therefore a progressive separation of the curves leading to an increase in the SER′.*

The precision of the SER′ depends on the precision of the estimates of X-ray doses to produce the same growth delay. Vertical growth-delay errors can be interpreted in terms of horizontal dose errors by drawing envelopes through the error bars. The standard error of the mean (s.e.) for the SER′ has been obtained from the fractional errors of the standard errors of the 2 doses. In these experiments the SER′

$$*SER = \frac{\text{dose X-rays alone}}{\text{dose X-rays with sensitizer}} \left\{ \begin{array}{l}\text{to achieve the same level of radiation}\\ \text{effect in fully hypoxic cells.}\end{array}\right.$$

SER′ = observed SER for a mixed population of oxic and hypoxic cells.

TABLE III.—*Pharmacology and toxicity*

| | Half-life in plasma (min)* | Effective volume of distribution (ml)* | Concentration at 20 min after 0·5 μmol/g i.v. | | | Concentration ratios 20 min after 0·5 μmol/g i.v. | | AUC brain (μmol/g.h)* | $LD_{50/7}$ | |
|---|---|---|---|---|---|---|---|---|---|---|
| | | | Blood | Tumour | Brain | Tumour/ Blood | Tumour/ Brain | | i.p. | (μmol/g) i.v. |
| MISO | 57 | 20 | 0·47 | 0·29 | 0·38 | 0·6 | 0·8 | 3·16 | 9·6 | >5 |
| Ro 05-9963 | 24 | 22 | 0·39 | 0·27 | 0·05 | 0·7 | 5·3 | 0·59 | 20·5 | >10 |
| Ro 03-8799 | 22† | 42† | 0·11 | 0·21 | 0·27 | 1·9 | 0·8 | 1·50§ | 6·1 | 2·4 |
| Ro 31-0052 | 19 | 35 | 0·13 | 0·15 | 0·02 | 1·2 | 8·8 | 0·15 | 10·0 | 4·9 |
| Ro 31-0054 | 12‡ | 154‡ | 0·08 | 0·10 | 0·11 | 1·3 | 0·9 | — | 0·8 | 0·7 |

* After i.v. injection of 2·5 μmol/g.

† After i.v. injection of 1·7 μmol/g.

‡ Data from Stratford *et al.* (1982) at 0·5 μmol/g.

§ By extrapolation from 1·7 μmol/g result.

estimates had a calculated standard error of ~10%.

Fig. 3 compares the measured tumour concentrations and the radiation response after i.v. injection of 4 of the compounds. Three of the drugs were administered at an equimolar dose of 2·5 μmol/g. Ro 03-8799 was administered at the maximum tolerated dose of 1·7 μmol/g. Ro 31-0054 is not shown because it was too toxic to test at comparable doses (i.v. $LD_{50} = 0 \cdot 7$ μmol/g). Fig. 3(a) shows the time course of drug concentrations in tumours, measured by HPLC. For all 4 drugs there is a plateau at 10–30 min; a similar broad peak at 5–20 min has been found after lower doses (0·5 μmol/g) of 4 of these compounds (excluding Ro 05-9963) (Stratford *et al.*, 1982).

Fig. 3(b) shows the corresponding radiobiological effect expressed as tumour growth delay for mice irradiated with 21·7 Gy at various intervals after i.v. administration of the drug. Zero time represents no drug administered. Increasing growth delay indicates radiosensitization. The maximum delay was seen at 10–20 min for all 4 compounds. Based on this assessment after high doses, a 20 min interval was used when comparing radiosensitizing activity over a range of drug doses.

MISO and Ro 03-8799 were also administered by the i.p. route for comparison with i.v. administration. Time-course studies were not performed in this work, but previous experiments have established that a 40 min interval between i.p. injection and the midpoint of irradiation achieves optimum radiosensitization in this tumour (Denekamp, 1982).

Fig. 4 shows the pharmacokinetics of the 4 compounds (*i.e.* excluding the toxic Ro 31-0054) in blood, brain and tumour. The longest half-life in blood was seen for MISO (57 min), which was distributed fairly uniformly throughout the body; by 20 min the tumour and brain concentrations were similar to those in blood. Tumour levels at 20 min were higher than those in blood for the basic compounds. Ro

FIG. 4.—Sensitizer concentrations measured by HPLC after i.v. injection of the indicated doses (μmol/g). The highest levels are obtained with MISO. Brain levels remain low with 9963 and 0052, but 8799 concentrates in brain and tumour. Half-lives are listed in Table III. ○ = blood; ● = tumour; △ = brain.

03-8799 was concentrated in brain, whereas Ro 05-9963 and Ro 31-0052 were *excluded*.

Table III summarizes the pharmacological and toxicity data. After i.v. administration the distribution phase is rapid, and the effective distribution volume has been calculated, assuming a 1-compartment model, by extrapolating the blood concentration curve back to zero time. Table III indicates effective volumes of distribution for MISO and Ro 05-9963 that are slightly less than the volume of the mouse, whereas with Ro 03-8799 and especially with Ro 31-0054 the effective volumes of distribution exceed that of the mice. This indicates rapid removal of the compound from the blood into other tissues.

The blood, tumour and brain levels 20 min afer a smaller dose of drug (0·5 μmol/g) are shown in Table III. The tumour concentrations of MISO and Ro 05-9963 are higher than those of the 3 charged compounds. The concentration in brain is highest for MISO and Ro 03-8799. Table III also shows tumour : brain ratios: this value (or alternatively the area under the concentration–time curve for brain) could indicate the therapeutic potential of the compound, if the limiting toxicity were only CNS damage. The tumour : brain ratio is inversely proportional to the distribution coefficient, as might be expected for brain exclusion of liphophobic compounds. The tumour:blood ratio of the basic compounds is higher than that for MISO and Ro 03-9963, and correlates with pKa. However, concentrations of Ro 03-8799 were also raised to the same degree in brain (Table III), fat, skeletal muscle, and liver (data not shown). The phenomenon is therefore one of concentration in all tissues rather than of selective uptake into tumours.

Toxicity was assessed by acute lethality (Table III). The mice were observed for 7 days, but death usually occurred within 2 days of i.p. administration, following a period of hypothermia. Because of the limitation placed on administered dose by restricting the injection volume to 0·4 ml, only a lower limit could be established for the i.v. $LD_{50}$ of MISO and Ro 05-9963. After i.v. administration of high doses of the basic compounds, those mice that died did so within minutes, after severe convulsions. Ro 05-9963 was the least toxic compound by either route of administration; Ro 31-0052 was similar in toxicity to MISO, whereas Ro 03-8799 was twice as toxic as MISO. There was no obvious correlation between toxicity and brain exposure or blood half-life, but Ro 31-0054 was the most toxic compound (10 times more than MISO) and it showed a similar increase in distribution volume; this may indicate rapid uptake into critical tissues.

Fig. 5 compares the radiosensitizing efficiency of the 5 compounds on the basis of equimolar doses. The line drawn by eye through the fibrosarcoma results for MISO in the first panel is reproduced as a standard for comparison on each subsequent graph. There is no significant difference between the results. Since the 3 basic compounds are of higher molecular weight than MISO, up to twice as much drug by weight is needed to give the same equimolar dose, and hence the same degree of radiosensitization. I.v. and i.p. admin-

FIG. 5.—SER′ measured by tumour growth delay as a function of the administered dose of sensitizer. The solid line drawn through the MISO results is reproduced as a broken line in other panels for comparison. None of the compounds is significantly better than MISO. △, i.p.; ●, iv.

FIG. 6.—SER′ for tumour growth delay as a function of measured gross tumour concentrations. The dashed lines represent the *in vitro* data from Fig. 1 for comparison. MISO and Ro 05-9963 appear marginally more effective than *in vitro*. The three basic compounds are significantly less effective than *in vitro*, especially at low concentrations. △, i.p.; ●, i.v.

istration of MISO and Ro 03-8799 gave similar results.

Radiosensitizing efficiency as a function of measured tumour concentration is shown in Fig. 6 and has been directly compared with the *in vitro* data from Fig. 1 (dashed lines). The *in vivo* points are fitted with a solid line. Slightly more sensitization of tumours was seen with MISO and Ro 05-9963 than would have been predicted from the *in vitro* results. The 3 basic compounds gave tumour sensitization intermediate between the 2 *in vitro* lines, showing greater efficiency than MISO, but less than that seen with V79 cells in culture. At high concentrations, Ro 03-8799 and Ro 31-0052 were up to 4 times more potent than MISO, but at the lower concentrations, *i.e.* 0·01–0·12 μmol/g, (which can be achieved clinically with 10–30 fractions of MISO) there was no significant difference between the 5 compounds.

### DISCUSSION

A radiosensitizer might be a clinical successor to misonidazole if it were either more potent or less toxic. The present studies were designed to compare, in concurrent experiments, the radiosensitizing efficiencies of 4 analogues of misonidazole with that of the parent compound. A wide range of drug doses was covered, from that which can be achieved clinically with MISO, to a dose approaching the toxic limit in mice. The optimum time for irradiation was determined for each compound at a single high dose level (1·7–2·5 μmol/g) as it can vary with the compound, the route of administration and type of tumour (McNally *et al.*, 1978; Brown & Yu, 1980). The sensitization obtained for each schedule was measured by comparing tumour responses at high levels of delay ($>25$ days in Fig. 2); naturally hypoxic cells then dominate the response, and the estimate of SER′ closely approaches the SER for a clamped, fully hypoxic tumour (Denekamp & Harris, 1975; Denekamp *et al.*, 1980*a*).

To ensure that an arbitrary choice of endpoint size did not bias the estimate of SER′ (Denekamp & Harris, 1975; Begg, 1980), the results were re-analysed using a range of endpoint sizes corresponding to a volume increase of 4–9-fold. Although it was found that tumours regrew at rates which varied with the growth delay induced by treatment, there was no significant difference in SER's measured at different endpoint sizes.

Two comparisons of the sensitizers were made: by administered dose and by tumour concentration. On the basis of equimolar dose, no difference was seen between the 5 compounds (Fig. 5). Pharmacokinetic differences mean that the basic compounds achieve lower tumour concentrations than MISO after equimolar dosage (Figs 3 & 4, Table III), and radiosensitizing efficiency was therefore compared on the basis of tumour concentration. Fig. 6 shows that the basic compounds were up to 4 times as potent as MISO. This is close to the prediction of a factor of 3 based on the physicochemical parameter electron-affinity, but is much less than the 10-fold increase in potency seen with V79 cells *in vitro* (Smithen *et al.*, 1980), a discrepancy which is explained below. At low concentrations, equivalent to those achieved clinically with MISO, the SER′ would only increase from 1·2 to 1·3; such a small difference would not be detectable in these experiments.

The accuracy of measurements of both drug concentration in tumours and the observed SER′ determines the reliability of estimates of relative efficiency of the 5 compounds. The large error bars in Fig. 3 show that the HPLC estimate of Ro 03-8799 concentration in individual tumours was more variable than for the other compounds. Because of this, and because of the clinical interest in Ro 03-8799, a complete repeat of this pharmacology experiment was performed; blood and tumour concentrations raised up to 2-fold. The reason for this greater variability with Ro 03-8799 is not understood, but the conclusions remain unchanged whichever set of data is used. The estimates of SER′

did not differ significantly in a repeat experiment (Figs 5 & 6) and the errors were certainly small enough to have detected the 10-fold difference in potency predicted from the *in vitro* data.

The discrepancy between the *in vivo* and *in vitro* results can be explained, because the concentration of a compound at its site of action is affected by its physicochemical and pharmacological properties. Both the *in vitro* data in Fig. 1 and the tumour data in Fig. 6 are expressed in terms of the gross concentration of drug, either in the medium surrounding the cells or in the homogenate made from tumours. Whilst these seem reasonable estimates, neither is a direct measure of intracellular drug concentration. Because charged molecules cross membranes relatively poorly (La Du *et al.*, 1971) the distribution of basic compounds is pH-dependent. This principle of ion trapping was one reason these compound were thought to have clinical potential (Wardman, 1982), both because they should be poorly reabsorbed from an acid urine, and because they might accumulate in the acidic regions shown to exist in tumours (Vaupel *et al.*, 1981). The 3 charged compounds have short half-lives as predicted, but the influence of pH on efficiency depends on whether the critical hypoxic cells themselves are internally acidic or whether there is simply a fall in extracellular pH. If there were concentration within hypoxic acidic cells in tumours, the basic compounds would be expected to be even more efficient in tumours than *in vitro*, which they clearly are not. Conversely, these drugs would accumulate extracellularly if the interstitial fluid were acidic but, unless such regions are a very lare proportion of the tumour, gross concentrations would closely parallel those in the critical hypoxic cells.

A pH-dependent change in the sensitizing efficiency of Ro 03-8799 *in vitro* has been reported for hypoxic V79 cells (Watts & Jones, 1981). The SER for 0·1 mM fell from 1·54 to 1·33 when the extracellular pH was reduced from 7·4 to 6·5. Recent measurements of intracellular concentrations of Ro 03-8799 as a function of extracellular pH have shown that this change in SER can be explained by changes in intracellular drug levels (Clarke *et al.*, 1982). Somewhat surprisingly, the concentration of Ro 03-8799 at pH 7·4 (at 25°C) was found to be twice that in the surrounding medium. With MISO, the intracellular concentration was only 50% of that in the medium. This differential concentration *in vitro* explains most, if not all, of the unexpectedly high efficiency of these basic compounds. Thus radiosensitizing efficiency measured *in vivo* agrees closely with that measured *in vitro*, when the basis of comparison is the intracellular concentration of each compound. The *in vitro* data, expressed in terms of the concentration in medium, were over-optimistic, and the 3-fold increase in efficiency predicted for these 3 basic compounds from electron-affinity fits both the corrected *in vitro* data and the present tumour results. This intracellular concentration of Ro 03-8799 at physiological pH would also explain the fact that the charged compounds achieved higher tumour:blood ratios than MISO at 20–30 min (Fig. 4, Table III). This concentration effect correlates with pKa (Table I) and was also observed in other tissues with Ro 03-8799.

The improvement in radiosensitizing efficiency which we have observed *in vivo* with the basic compounds is slight, and any substantial clinical advantage would depend on reduced toxicity in man (*i.e.* the ability to administer a total of $>12$ g/m$^2$ during a course of radiotherapy). Our toxicity data are limited to acute lethality and sensitizer concentrations in mouse brains. Table III shows that Ro 03-8799 is more toxic than MISO in mice, when judged by lethality, but less toxic if judged by the AUC for brain; Ro 05-9963 is the least toxic of all and Ro 31-0052 is the least toxic of the 3 basic compounds. However, this assessment bears no direct relationship to the presumptive limiting toxicity in man: peripheral neurotoxicity.

Brown & Workman (1980) have suggested that neurotoxicity might be reduced by lowering the lipophilicity of compounds by structural modification, in order to reduce drug entry into nervous tissue. As they predict, brain exposure (assessed on the basis of AUC) is correlated with distribution coefficient (Tables I & III) and Ro 31-0052 has the lowest value. This may well relate to central neuropathy, but does not seem to predict for clinical peripheral neuropathy, for the following reason: although the AUC value for Ro 05-9963 is one fifth that for MISO in the mouse (Table III), we know that these 2 drugs are equally toxic for peripheral neuropathy in man (Dische *et al.*, 1981; Coleman *et al.*, 1982). Unfortunately, no laboratory assay in rodents has correctly ranked MISO, Ro 05-9963 and metronidazole, the neurotoxic dose limits of which have all been established in man (Hirst *et al.*, 1979; Clarke *et al.*, 1980; Conroy *et al.*, 1980; Brown *et al.*, 1981). The selection of compounds for extensive animal toxicology before clinical trial therefore remains arbitrary.

The toxicity of Ro 03-8799 has been comprehensively examined in rats and monkeys, with daily i.v. injections over 4 weeks (M. R. Jackson, personal communication). The results with *Cynomolgus* monkeys, when compared with previous experience with MISO in this species, show that considerably larger doses of Ro 03-8799 are tolerated, the limiting toxicity being liver damage rather than neurotoxicity. This finding has encouraged Phase I clinical trials to be undertaken with Ro 03-8799 (Dische *et al.*, 1982).

The overall enhancement seen with MISO or any potential successor will be much less in clinical use than in the results obtained with large single doses of drug and X-rays in mice. Any reoxygenation will markedly diminish the gain there might otherwise be. The use of small X-ray doses per fraction will further reduce the observed SER′, because oxic cells will dominate the response unless reoxygenation is very poor (Denekamp *et al.*, 1980*b*; Durand & Olive, 1981; Denekamp & Joiner, 1982). Nevertheless, sensitization has been found in human tumours treated with 9–10 fractions of irradiation (Urtasun *et al.*, 1977; Ash *et al.*, 1979). The presence of hypoxic cells in human tumours is now well documented, and their persistence for at least 10 fractions in some tumours is indicated.

Before randomized clinical trials of a new compound to replace MISO are initiated, a marked gain in sensitization needs to be demonstrated *in vivo*. The present results are consistent with a 3–4-fold increase in potency of the basic compounds relative to MISO, but at low, clinically relevant, concentrations, the improvement in SER′ one would then expect is very small, and will be less still if the pharmacokinetics in mouse and man are similar (*i.e.* if tumour levels of Ro 03-8799 are lower than those after an equimolar dose of MISO). Any substantial advantage would depend on reduced toxicity in man; the primate toxicology results obtained with Ro 03-8799 are encouraging in this respect.

This research was financed by the Cancer Research Campaign. We are grateful to Roche Products Ltd, Welwyn Garden City, for providing all the compounds tested, and for the unpublished toxicology data on Ro 03-8799. We thank Mr P. Russell and his staff for provision and care of the animals; Dr M. E. Watts for the data from which Fig. 1 was drawn; Mr N. H. A. Terry for the i.p. $LD_{50}$ data; Dr A. G. Allen and Dr C. E. Smithen for their constructive criticism of the final manuscript; and Mrs G. Mason and Mrs E. Marriott for their secretarial assistance.

### REFERENCES

ADAMS, G. E., FLOCKHART, I. R., SMITHEN, C. E., STRATFORD, I. J., WARDMAN, P. & WATTS, M. E. (1976) Electron-affinic sensitization. VII. A correlation between structures, one-electron reduction potentials and efficiencies of nitroimidazoles as hypoxic cell radiosensitizers. *Radiat. Res.*, **670**, 9.

ASH, D. V., PECKHAM, M. J. & STEEL, G. G. (1979) The quantitative response of human tumours to radiation and misonidazole. *Br. J. Cancer*, **40**, 883.

BEGG, A. C. (1980) Analysis of growth delay: Potential pitfalls. *Br. J. Cancer*, **41**, (Suppl. IV), 93.

BROWN, J. M. & WORKMAN, P. (1980) Partition coefficient as a guide to the development of radiosensitizers which are less toxic than misonidazole. *Radiat. Res.*, **82**, 171.

BROWN, J. M. & YU, N. Y. (1980) The optimum time for irradiation relative to tumour concentration of hypoxic cell sensitizers. *Br. J. Radiol.*, **53**, 915.

BROWN, J. M., YU, N. Y., BROWN, D. M. & LEE, W. W. (1981) SR-2508: A 2-nitroimidazole amide which should be superior to misonidazole as a radiosensitizer for clinical use. *Int. J. Radiat. Oncol. Biol. Phys.*, **7**, 695.

CLARKE, C., DAWSON, K. B. & SHELDON, P. W. (1980) Quantitative cytochemical method for assessing the neurotoxicity of misonidazole. In *Radiation Sensitizers: Their Use in the Clinical Management of Cancer.* (Ed. Brady). New York: Masson. p. 245.

CLARKE, E. D., DENNIS, M. F., JONES, N. R. & 4 others (1982) The importance of pH-induced concentration gradients in the use of nitroimidazole radiosensitizers with basic and acidic substituents. *Br. J. Radiol.* (in press).

COLEMAN, C. N., WASSERMAN, T. H., PHILLIPS, T. L. & 4 others (1982) Initial pharmacology and toxicology of desmethylmisonidazole. *Int. J. Radiat. Oncol. Biol. Phys.*, **8**, 371.

CONROY, P. J., SHAW, A. B., MCNEILL, T. H., PASCALACQUA, W. & SUTHERLAND, R. M. (1980) Radiation sensitizer neurotoxicity in the mouse. In *Radiation Sensitizers: Their Use in the Clinical Management of Cancer* (Ed. Brady). New York: Masson. p. 397.

DENEKAMP, J. (1982) Biological methods for studying radiosensitization. In *Advanced Topics in Radiosensitizers of Hypoxic Cells.* (Eds. Adams *et al.*). New York: Plenum Press. p. 119.

DENEKAMP, J. & HARRIS, S. R. (1975) Tests of two electron-affinic radiosensitizers using regrowth of an experimental carcinoma. *Radiat. Res.*, **61**, 191.

DENEKAMP, J., HIRST, D. G., STEWART, F. A. & TERRY, N. H. A. (1980*a*) Tumour radiosensitization by misonidazole: A general phenomenon? *Br. J. Cancer*, **41**, 1.

DENEKAMP, J. & JOINER, M. C. (1982) The potential benefit from a perfect radiosensitizer and its dependence on reoxygenation. *Br. J. Radiol.*, **5** (in press).

DENEKAMP, J., MCNALLY, N. J., FOWLER, J. F. & JOINER, M. C. (1980*b*) Misonidazole in fractionated radiotherapy: Are many small fractions best? *Br. J. Radiol.*, **53**, 981.

DISCHE, S., SAUNDERS, M. I., ANDERSON, P., STRATFORD, M. R. L. & MINCHINTON, A. (1982) Clinical experience with nitroimidazoles as radiosensitizers. *Int. J. Radiat. Oncol. Biol. Phys.*, **8**, 335.

DISCHE, S., SAUNDERS, M. I., FLOCKHART, I. R., LEE, M. E. & ANDERSON, P. (1979) Misonidazole—A drug for trial in radiotherapy and oncology. *Int. J. Radiat. Oncol. Biol. Phys.*, **5**, 851.

DISCHE, S., SAUNDERS, M. I. & STRATFORD, M. R. L. (1981) Neurotoxicity with desmethylmisonidazole. *Br. J. Radiol.*, **54**, 156.

DURAND, R. E. & OLIVE, P. L. (1981) Evaluation of nitroheterocyclic radiosensitizers using spheroids. *Adv. Radiat. Biol.*, **9**, 75.

FOWLER, J. F. & DENEKAMP, J. (1979) A review of hypoxic cell radiosensitization in experimental tumours. *Pharmacol. Ther.*, **7**, 413.

FOWLER, J. F., SHELDON, P. W., BEGG, A. C., HILL, S. A. & SMITH, A. M. (1975) Biological properties and response to X-rays of first-generation transplants of spontaneous mammary carcinomas in C3H mice. *Int. J. Radiat. Biol.*, **27**, 463.

HIRST, D. G., VOJNOVIC, B. & HOBSON, B. (1979) Changes in nerve conduction velocity in the mouse after acute and chronic administration of nitroimidazoles. *Br. J. Cancer*, **39**, 159.

LA DU, B. V., MANDEL, H. G. & WAY, E. L. (1971) *Fundamentals of Drug Metabolism and Drug Disposition.* Baltimore: Masson.

MCNALLY, N. J., DENEKAMP, J., SHELDON P., FLOCKHART, I. R. & STEWART, F. A. (1978) Radiosensitization by misonidazole (Ro 07-0582): The importance of timing and tumour concentration of sensitizer. *Radiat. Res.* **73**, 568.

SHELDON, P. W. & HILL, S. A. (1977) Hypoxic cell radiosensitizers and local control by X-ray of a transplanted tumour in mice. *Br. J. Cancer* **35**, 795.

SMITHEN, C. E., WARDMAN, P., CLARKE, E. D. & 4 others (1980) Novel (nitro-1-imidazolyl)-alkanolamines as potential radiosensitizers with improved therapeutic properties. In *Radiation Sensitizers: Their Use in the Clinical Management of Cancer.* (Ed. Brady). New York: Masson. p. 22.

STRATFORD, M. R. L., MINCHINTON, A. I., STEWART, F. A. & RANDHAWA, V. S. (1982) Pharmacokinetic studies on some novel (2-nitro-1-imidazolyl) propanolamine radiosensitizers. In *Advanced Topics on Radiosensitizers of Hypoxic Cells.* (Eds. Adams *et al.*). New York: Plenum Press p. 165.

THOMLINSON, R. H. & CRADDOCK, E. A. (1967) The gross response of an experimental tumour to single doses of X-rays. *Br. J. Cancer* **21**, 108.

URTASUN, R. C., CHAPMAN, J. D., FELDSTEIN, M. L. & 6 others (1978) Peripheral neuropathy related to misonidazole: Incidence and pathology. *Br. J. Cancer*, **37** (Suppl. III), 271.

URTASUN, R. C., CHAPMAN, J. D., BAND, P., RABIN, H. R., FRYER, C. G. & STURMWIND, J. (1975) Phase I study of high-dose metronidazole: A specific *in vivo* and *in vitro* radiosensitizer of hypoxic cells. *Radiology*, **117**, 129.

URTASUN, R. C., MILLER, J. D. R., FRUNCHAK, V. & KOZIOL, D. (1977) Radiotherapy pilot trials with sensitizers of hypoxic cells: Metronidazole in supratentorial glioblastomas. *Br. J. Radiol.*, **50**, 602.

VAUPEL, P. W., FRINAK, S. & BICHER, H. I. (1981) Heterogeneous oxygen partial pressure and pH distribution in C3H mouse mammary adenocarcinoma. *Cancer Res.*, **41**, 2008.

WARDMAN, P. (1982) Molecular structure and biological activity of hypoxic cell radiosensitizers and hypoxic-specific cytotoxins. In *Advanced Topics in Radiosensitizers of Hypoxic Cells.* (Eds. Adams *et al.*). New York: Plenum Press. p. 49.

WASSERMAN, T. H., PHILLIPS, T. L., JOHNSON, R. J. & 6 others (1979) Initial United States clinical and pharmacologic evaluation of misonidazole (Ro 07-0582), an hypoxic cell radiosensitizer. *Int. J. Radiat. Oncol. Biol. Phys.*, **5**, 776.

WATTS, M. E., ANDERSON, R. F., JACOBS, R. S. & 7 others (1980) Evaluation of novel hypoxic cell radiosensitizers *in vitro*. The value of single cell systems. In *Radiation Sensitizers: Their Use in the Clinical Management of Cancer.* (Ed. Brady). New York: Masson. p. 175.

WATTS, M. E. & JONES, N. R. (1981) Radiosensitization of hypoxic mammalian cells by nitroimidazoles with acidic or basic substituents: The effects of extracellular pH. *Radiat. Res.*, **87**, 479.